# A trinuclear cobalt–cerium complex: bis­(2,2′-bipyridine)-1κ^2^ *N*,*N*′;3κ^2^ *N*,*N*′-hexa-μ-methacrylato-1:2κ^6^ *O*:*O*′;2:3κ^6^ *O*:*O*′-nitrato-2κ^2^ *O*,*O*′-2-cerium(III)-1,3-dicobalt(II)

**DOI:** 10.1107/S1600536810010299

**Published:** 2010-03-27

**Authors:** Bin Wu, Tingting Hou

**Affiliations:** a Department of Applied Chemistry, Zhejiang Sci-Tech University, Hangzhou 310018, People’s Republic of China

## Abstract

In the title trinuclear cobalt-cerium complex, [CeCo_2_(C_4_H_5_O_2_)_6_(NO_3_)(C_10_H_8_N_2_)_2_], the Ce^III^ and each of the two Co^II^ ions are bridged by three bidentate methacrylate ligands. The Ce^III^ center is coordinated by six O atoms from six methacrylate ligands and two O atoms from the nitrate anion in a distorted square-anti­prismatic geometry. Each Co^II^ ion is coordinated by three O atoms from three methacrylate ligands and two N atoms from a 2,2′-bipyridine ligand in a distorted trigonal-pyramidal geometry. In the crystal structure, π–π inter­actions between the aromatic rings [centroid–centroid distances of 3.816 (8) and 3.756 (8) Å] link the mol­ecules into chains propagated in [01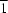]. Weak inter­molecular C—H⋯O hydrogen bonds further stabilize the crystal packing.

## Related literature

For the crystal structures of analogous complexes, see: Wu & Guo (2004 ▶); Zhu *et al.* (2004*a* ▶,*b* ▶; 2005 ▶). For the preparation of Ce*L*_3_·2H_2_O (H*L* = CH_2_C(CH_3_)COOH), see: Lu *et al.* (1995 ▶). 
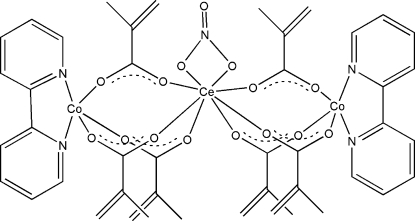


## Experimental

### 

#### Crystal data

[CeCo_2_(C_4_H_5_O_2_)_6_(NO_3_)(C_10_H_8_N_2_)_2_]
*M* *_r_* = 1142.84
Triclinic, 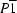

*a* = 11.4445 (8) Å
*b* = 13.6484 (9) Å
*c* = 16.5051 (10) Å
α = 104.108 (9)°
β = 99.937 (8)°
γ = 100.115 (7)°
*V* = 2398.0 (3) Å^3^
*Z* = 2
Mo *K*α radiation
μ = 1.69 mm^−1^
*T* = 292 K
0.35 × 0.30 × 0.28 mm

#### Data collection

Rigaku R-AXIS RAPID diffractometer
Absorption correction: multi-scan (*ABSCOR*; Higashi, 1995 ▶) *T*_min_ = 0.559, *T*_max_ = 0.623
17512 measured reflections
8121 independent reflections
7037 reflections with *I* > 2σ(*I*)
*R*_int_ = 0.020

#### Refinement

*R*[*F* ^2^ > 2σ(*F* ^2^)] = 0.023
*wR*(*F* ^2^) = 0.054
*S* = 1.03
8121 reflections
610 parameters
H-atom parameters constrained
Δρ_max_ = 0.27 e Å^−3^
Δρ_min_ = −0.36 e Å^−3^

### 

Data collection: *RAPID-AUTO* (Rigaku, 1998 ▶); cell refinement: *RAPID-AUTO*; data reduction: *CrystalStructure* (Rigaku/MSC, 2002 ▶); program(s) used to solve structure: *SHELXS97* (Sheldrick, 2008 ▶); program(s) used to refine structure: *SHELXL97* (Sheldrick, 2008 ▶); molecular graphics: *ORTEP-3* (Farrugia, 1997 ▶); software used to prepare material for publication: *SHELXL97* and *PLATON* (Spek, 2009 ▶).

## Supplementary Material

Crystal structure: contains datablocks global, I. DOI: 10.1107/S1600536810010299/cv2700sup1.cif

Structure factors: contains datablocks I. DOI: 10.1107/S1600536810010299/cv2700Isup2.hkl

Additional supplementary materials:  crystallographic information; 3D view; checkCIF report

## Figures and Tables

**Table 1:** Hydrogen-bond geometry (Å, °)

*D*—H⋯*A*	*D*—H	H⋯*A*	*D*⋯*A*	*D*—H⋯*A*
C2—H2⋯O7^i^	0.93	2.59	3.322 (4)	136
C3—H3⋯O9^i^	0.93	2.45	3.307 (5)	154
C7—H7⋯O3^ii^	0.93	2.58	3.452 (4)	156
C37—H37⋯O9^iii^	0.93	2.51	3.364 (4)	152

Symmetry codes: (i) 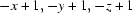; (ii) 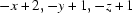; (iii) 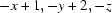.

**Fig. 1.**
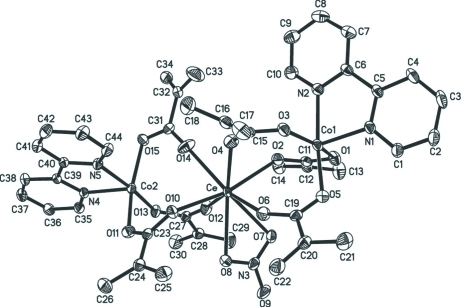
View of the title molecule showing the atom-labelling scheme. Displacement ellipsoids are drawn at the 30% probability level. All H atoms have been omitted for clarity.

## supplementary crystallographic information

### Comment

As a contribution to a structural study of heterometallic complexes containing d-transition metal and rare-earth(III) cations (Wu & Guo, 2004; Zhu *et al.*, 2004a,b; Zhu *et al.*, 2005), herewith we report the synthesis and crystal structure of the title compound.

The crystal structure of the title Co—Ce—Co trinuclear complex is similar to the known crystal structures of the Zn—Nd—Zn, Zn—Pr—Zn, Zn—La—Zn and Zn—Ce—Zn complexes (Wu & Guo, 2004; Zhu *et al.*, 2004a,b; Zhu *et al.*, 2005). The Ce^III^ center is coordinated by six O atoms from six methacrylato ligands and two O atoms from nitrate anion in a distorted square-antiprismatic geometry. Each Co^II^ ion is coordinated by three O atoms from three methacrylato ligands and two N atoms from 2,2'-bipyridine ligand in a distorted pyramidal geometry. The Ce^III^ and each of two Co^II^ ions are bridged by three bidentate methacrylato ligands. Two Ce···Co separations are almost equal being 3.944 (1) and 3.993 (1) Å, respectively.

In the crystal structure, π-π interactions between the aromatic rings (Table 1) link molecules into chains propagated in direction [01-1]. Weak intermolecular C—H···O hydrogen bonds (Table 2) stabilize further the crystal packing.

### Experimental

CeL_3_.2H_2_O (870 mg, 2.0 mmol; HL = CH_2_C(CH_3_)COOH ) prepared in accordance with Lu *et al.* (1995) and Co(NO_3_)_2_.6H_2_O (435 mg, 1.5 mmol) were dissolved in 15 ml H_2_O, and the pH adjusted to 4.0 using HL. Three mililiters of ethanol solution 2,2'-bipyridine (234 mg, 1.5 mmol) were added into the mixed solution with stirring. After filtration, the filtrate was allowed to stand at room temperature and single crystals suitable for X-ray work were obtained after two weeks.

### Refinement

All H-atoms were placed in idealized locations with C–H distances 0.93 - 0.96 Å and refined as riding with  U_iso_(H) = 1.2 or 1.5 U_iso_(C).

### Crystal data

**Table:**

[CeCo_2_(C_4_H_5_O_2_)_6_(NO_3_)(C_10_H_8_N_2_)_2_]	*Z* = 2
*M**_r_* = 1142.84	*F*(000) = 1154
Triclinic, *P*1	*D*_x_ = 1.583 Mg m^−^^3^
Hall symbol: -P 1	Mo *K*α radiation, λ = 0.71069 Å
*a* = 11.4445 (8) Å	Cell parameters from 7585 reflections
*b* = 13.6484 (9) Å	θ = 2.3–27.4°
*c* = 16.5051 (10) Å	µ = 1.69 mm^−^^1^
α = 104.108 (9)°	*T* = 292 K
β = 99.937 (8)°	Prism, brown
γ = 100.115 (7)°	0.35 × 0.30 × 0.28 mm
*V* = 2398.0 (3) Å^3^	

### Data collection

**Table:**

Rigaku R-AXIS RAPID diffractometer	8121 independent reflections
Radiation source: fine-focus sealed tube	7037 reflections with *I* > 2σ(*I*)
graphite	*R*_int_ = 0.020
Detector resolution: 10.00 pixels mm^-1^	θ_max_ = 25.1°, θ_min_ = 1.3°
ω scans	*h* = −13→13
Absorption correction: multi-scan (*ABSCOR*; Higashi, 1995)	*k* = −16→16
*T*_min_ = 0.559, *T*_max_ = 0.623	*l* = −19→19
17512 measured reflections	

### Refinement

**Table:**

Refinement on *F*^2^	Primary atom site location: structure-invariant direct methods
Least-squares matrix: full	Secondary atom site location: difference Fourier map
*R*[*F*^2^ > 2σ(*F*^2^)] = 0.023	Hydrogen site location: inferred from neighbouring sites
*wR*(*F*^2^) = 0.054	H-atom parameters constrained
*S* = 1.03	*w* = 1/[σ^2^(*F*_o_^2^) + (0.0302*P*)^2^ + 0.5473*P*] where *P* = (*F*_o_^2^ + 2*F*_c_^2^)/3
8121 reflections	(Δ/σ)_max_ = 0.002
610 parameters	Δρ_max_ = 0.27 e Å^−^^3^
0 restraints	Δρ_min_ = −0.36 e Å^−^^3^

### Special details

**Table:**

Geometry. All esds (except the esd in the dihedral angle between two l.s. planes) are estimated using the full covariance matrix. The cell esds are taken into account individually in the estimation of esds in distances, angles and torsion angles; correlations between esds in cell parameters are only used when they are defined by crystal symmetry. An approximate (isotropic) treatment of cell esds is used for estimating esds involving l.s. planes.
Refinement. Refinement of *F*^2^ against ALL reflections. The weighted *R*-factor wR and goodness of fit *S* are based on *F*^2^, conventional *R*-factors *R* are based on *F*, with *F* set to zero for negative *F*^2^. The threshold expression of *F*^2^ > σ(*F*^2^) is used only for calculating *R*-factors(gt) etc. and is not relevant to the choice of reflections for refinement. *R*-factors based on *F*^2^ are statistically about twice as large as those based on *F*, and *R*- factors based on ALL data will be even larger.

### Fractional atomic coordinates and isotropic or equivalent isotropic displacement parameters (Å^2^)

**Table:**

	*x*	*y*	*z*	*U*_iso_*/*U*_eq_	
Ce	0.646568 (11)	0.762318 (11)	0.257303 (8)	0.03641 (5)	
Co1	0.71543 (3)	0.57517 (3)	0.39854 (2)	0.04127 (8)	
Co2	0.71375 (3)	0.90602 (2)	0.080876 (19)	0.03731 (8)	
O1	0.56474 (17)	0.46603 (15)	0.31540 (12)	0.0602 (5)	
O2	0.62793 (16)	0.57970 (14)	0.25067 (13)	0.0581 (5)	
O3	0.86748 (15)	0.69345 (14)	0.44496 (12)	0.0544 (4)	
O4	0.84746 (16)	0.75916 (17)	0.33430 (12)	0.0640 (5)	
O5	0.62048 (16)	0.66522 (16)	0.46295 (12)	0.0569 (5)	
O6	0.64355 (18)	0.79847 (17)	0.40766 (11)	0.0642 (5)	
O7	0.42302 (17)	0.68799 (17)	0.26372 (13)	0.0634 (5)	
O8	0.46456 (18)	0.85157 (17)	0.27586 (14)	0.0652 (5)	
O9	0.2814 (2)	0.7743 (2)	0.27262 (18)	0.1017 (10)	
O10	0.71556 (17)	0.94278 (15)	0.27044 (15)	0.0679 (6)	
O11	0.66255 (16)	1.01476 (15)	0.16728 (12)	0.0561 (5)	
O12	0.52216 (16)	0.73813 (17)	0.11668 (12)	0.0634 (5)	
O13	0.54427 (14)	0.82487 (14)	0.02171 (11)	0.0489 (4)	
O14	0.78294 (19)	0.7574 (2)	0.15643 (13)	0.0843 (7)	
O15	0.80120 (15)	0.79025 (14)	0.03581 (12)	0.0525 (4)	
N1	0.73679 (17)	0.49011 (16)	0.48701 (13)	0.0459 (5)	
N2	0.83532 (18)	0.48188 (17)	0.35382 (13)	0.0484 (5)	
N3	0.3877 (2)	0.7720 (2)	0.27117 (16)	0.0640 (7)	
N4	0.73331 (16)	0.98929 (15)	−0.00789 (12)	0.0397 (4)	
N5	0.89352 (17)	1.00305 (16)	0.13205 (12)	0.0414 (5)	
C1	0.6747 (2)	0.4913 (2)	0.54883 (18)	0.0581 (7)	
H1	0.6199	0.5340	0.5544	0.070*	
C2	0.6889 (3)	0.4319 (3)	0.6042 (2)	0.0711 (9)	
H2	0.6437	0.4338	0.6461	0.085*	
C3	0.7705 (3)	0.3699 (3)	0.5972 (2)	0.0785 (10)	
H3	0.7829	0.3302	0.6350	0.094*	
C4	0.8341 (3)	0.3668 (2)	0.5334 (2)	0.0663 (8)	
H4	0.8893	0.3244	0.5272	0.080*	
C5	0.8149 (2)	0.4277 (2)	0.47851 (16)	0.0470 (6)	
C6	0.8746 (2)	0.42659 (19)	0.40572 (16)	0.0459 (6)	
C7	0.9639 (3)	0.3721 (2)	0.3901 (2)	0.0651 (8)	
H7	0.9916	0.3353	0.4271	0.078*	
C8	1.0106 (3)	0.3733 (3)	0.3193 (2)	0.0779 (10)	
H8	1.0700	0.3369	0.3076	0.093*	
C9	0.9693 (3)	0.4285 (3)	0.2660 (2)	0.0771 (10)	
H9	0.9995	0.4293	0.2173	0.092*	
C10	0.8826 (3)	0.4828 (3)	0.28554 (19)	0.0637 (8)	
H10	0.8560	0.5215	0.2499	0.076*	
C11	0.5575 (2)	0.4969 (2)	0.24968 (17)	0.0456 (6)	
C12	0.4677 (2)	0.4326 (2)	0.16978 (16)	0.0487 (6)	
C13	0.3860 (3)	0.3415 (3)	0.1760 (2)	0.0865 (11)	
H13A	0.3362	0.3627	0.2152	0.130*	
H13B	0.4331	0.2971	0.1965	0.130*	
H13C	0.3350	0.3045	0.1205	0.130*	
C14	0.4674 (3)	0.4585 (3)	0.09614 (19)	0.0747 (9)	
H14A	0.4131	0.4172	0.0458	0.090*	
H14B	0.5215	0.5176	0.0956	0.090*	
C15	0.8992 (2)	0.7601 (2)	0.40809 (16)	0.0453 (6)	
C16	1.0064 (2)	0.8472 (2)	0.4549 (2)	0.0555 (7)	
C17	1.0254 (3)	0.8825 (3)	0.5413 (2)	0.0812 (10)	
H17A	1.0899	0.9383	0.5717	0.097*	
H17B	0.9740	0.8511	0.5701	0.097*	
C18	1.0817 (3)	0.8911 (3)	0.4038 (3)	0.0993 (13)	
H18A	1.1406	0.9517	0.4400	0.149*	
H18B	1.0310	0.9096	0.3599	0.149*	
H18C	1.1230	0.8407	0.3777	0.149*	
C19	0.6267 (2)	0.7587 (2)	0.46692 (16)	0.0463 (6)	
C20	0.6162 (2)	0.8285 (2)	0.54997 (16)	0.0523 (7)	
C21	0.6097 (3)	0.7816 (3)	0.62045 (19)	0.0818 (10)	
H21A	0.6089	0.8338	0.6712	0.123*	
H21B	0.6793	0.7525	0.6312	0.123*	
H21C	0.5367	0.7279	0.6052	0.123*	
C22	0.6135 (3)	0.9272 (3)	0.5566 (2)	0.0879 (11)	
H22A	0.6073	0.9703	0.6080	0.106*	
H22B	0.6179	0.9530	0.5097	0.106*	
C23	0.6811 (2)	1.0143 (2)	0.24425 (18)	0.0467 (6)	
C24	0.6657 (2)	1.1069 (2)	0.30774 (16)	0.0482 (6)	
C25	0.6632 (3)	1.1034 (3)	0.3872 (2)	0.0760 (9)	
H25A	0.6522	1.1605	0.4268	0.091*	
H25B	0.6725	1.0439	0.4031	0.091*	
C26	0.6518 (3)	1.1977 (2)	0.2777 (2)	0.0721 (8)	
H26A	0.6450	1.2526	0.3244	0.108*	
H26B	0.5797	1.1796	0.2326	0.108*	
H26C	0.7216	1.2205	0.2563	0.108*	
C27	0.4831 (2)	0.76085 (19)	0.05019 (15)	0.0411 (5)	
C28	0.3557 (2)	0.7090 (2)	0.00004 (17)	0.0517 (6)	
C29	0.2859 (3)	0.6420 (3)	0.0310 (3)	0.0886 (12)	
H29A	0.2059	0.6105	0.0019	0.106*	
H29B	0.3182	0.6278	0.0815	0.106*	
C30	0.3137 (3)	0.7356 (3)	−0.0770 (2)	0.0765 (9)	
H30A	0.3527	0.7050	−0.1206	0.115*	
H30B	0.3333	0.8097	−0.0659	0.115*	
H30C	0.2271	0.7100	−0.0960	0.115*	
C31	0.8207 (2)	0.7411 (2)	0.08925 (16)	0.0455 (6)	
C32	0.8997 (3)	0.6651 (2)	0.0737 (2)	0.0587 (7)	
C33	0.9661 (4)	0.6462 (4)	0.1427 (3)	0.1169 (16)	
H33A	1.0190	0.6020	0.1348	0.140*	
H33B	0.9586	0.6776	0.1977	0.140*	
C34	0.9059 (3)	0.6198 (2)	−0.0135 (2)	0.0739 (9)	
H34A	0.9546	0.5691	−0.0148	0.111*	
H34B	0.9421	0.6731	−0.0362	0.111*	
H34C	0.8253	0.5870	−0.0475	0.111*	
C35	0.6458 (2)	0.9808 (2)	−0.07628 (17)	0.0500 (6)	
H35	0.5707	0.9359	−0.0846	0.060*	
C36	0.6625 (3)	1.0360 (2)	−0.13475 (18)	0.0587 (7)	
H36	0.5999	1.0290	−0.1814	0.070*	
C37	0.7738 (3)	1.1013 (2)	−0.12245 (19)	0.0616 (8)	
H37	0.7881	1.1388	−0.1613	0.074*	
C38	0.8641 (2)	1.1111 (2)	−0.05225 (17)	0.0512 (6)	
H38	0.9400	1.1550	−0.0434	0.061*	
C39	0.8415 (2)	1.05532 (18)	0.00512 (15)	0.0390 (5)	
C40	0.9312 (2)	1.06372 (18)	0.08404 (15)	0.0392 (5)	
C41	1.0465 (2)	1.1292 (2)	0.10949 (17)	0.0508 (6)	
H41	1.0728	1.1696	0.0753	0.061*	
C42	1.1214 (2)	1.1339 (2)	0.18549 (18)	0.0585 (7)	
H42	1.1983	1.1783	0.2035	0.070*	
C43	1.0824 (2)	1.0729 (2)	0.23502 (18)	0.0584 (7)	
H43	1.1317	1.0752	0.2869	0.070*	
C44	0.9683 (2)	1.0083 (2)	0.20546 (16)	0.0520 (6)	
H44	0.9418	0.9660	0.2382	0.062*	

### Atomic displacement parameters (Å^2^)

**Table:**

	*U*^11^	*U*^22^	*U*^33^	*U*^12^	*U*^13^	*U*^23^
Ce	0.03874 (8)	0.04090 (9)	0.03799 (8)	0.01186 (5)	0.01074 (5)	0.02308 (6)
Co1	0.04147 (17)	0.0443 (2)	0.04137 (19)	0.01198 (14)	0.00418 (14)	0.02014 (15)
Co2	0.03676 (16)	0.03833 (19)	0.03931 (18)	0.00528 (13)	0.00993 (13)	0.01688 (14)
O1	0.0639 (11)	0.0597 (13)	0.0508 (11)	0.0093 (9)	−0.0065 (9)	0.0215 (10)
O2	0.0504 (10)	0.0411 (11)	0.0814 (14)	0.0080 (8)	0.0158 (9)	0.0157 (10)
O3	0.0470 (10)	0.0486 (11)	0.0671 (12)	0.0086 (8)	0.0019 (9)	0.0250 (10)
O4	0.0488 (10)	0.0902 (16)	0.0533 (12)	0.0172 (10)	0.0020 (9)	0.0263 (11)
O5	0.0580 (11)	0.0572 (13)	0.0633 (12)	0.0199 (9)	0.0213 (9)	0.0213 (10)
O6	0.0829 (14)	0.0824 (15)	0.0441 (11)	0.0366 (11)	0.0239 (10)	0.0291 (10)
O7	0.0554 (11)	0.0738 (15)	0.0812 (14)	0.0163 (10)	0.0258 (10)	0.0505 (12)
O8	0.0638 (12)	0.0733 (15)	0.0840 (15)	0.0334 (11)	0.0304 (11)	0.0468 (12)
O9	0.0588 (14)	0.170 (3)	0.139 (2)	0.0587 (15)	0.0532 (14)	0.112 (2)
O10	0.0612 (12)	0.0474 (12)	0.1085 (17)	0.0146 (9)	0.0286 (11)	0.0381 (12)
O11	0.0548 (11)	0.0583 (13)	0.0511 (12)	0.0120 (9)	0.0141 (9)	0.0072 (9)
O12	0.0534 (11)	0.0855 (15)	0.0514 (12)	0.0028 (10)	0.0014 (9)	0.0359 (11)
O13	0.0425 (9)	0.0517 (11)	0.0494 (10)	−0.0020 (8)	0.0051 (8)	0.0212 (9)
O14	0.0600 (12)	0.145 (2)	0.0551 (12)	0.0286 (13)	0.0293 (10)	0.0264 (13)
O15	0.0555 (10)	0.0451 (11)	0.0603 (12)	0.0157 (8)	0.0117 (9)	0.0189 (9)
N1	0.0424 (11)	0.0491 (13)	0.0478 (12)	0.0083 (9)	0.0031 (9)	0.0233 (10)
N2	0.0480 (12)	0.0488 (14)	0.0487 (13)	0.0124 (10)	0.0083 (10)	0.0154 (10)
N3	0.0511 (14)	0.102 (2)	0.0688 (16)	0.0322 (14)	0.0265 (12)	0.0587 (15)
N4	0.0409 (10)	0.0391 (12)	0.0434 (11)	0.0092 (8)	0.0101 (9)	0.0190 (9)
N5	0.0404 (10)	0.0446 (12)	0.0391 (11)	0.0048 (9)	0.0079 (9)	0.0163 (9)
C1	0.0508 (15)	0.070 (2)	0.0617 (18)	0.0106 (13)	0.0121 (13)	0.0353 (15)
C2	0.0696 (19)	0.084 (2)	0.071 (2)	0.0069 (17)	0.0211 (16)	0.0457 (18)
C3	0.094 (2)	0.076 (2)	0.080 (2)	0.0159 (19)	0.0125 (19)	0.056 (2)
C4	0.0725 (19)	0.0546 (19)	0.078 (2)	0.0197 (15)	0.0030 (16)	0.0367 (16)
C5	0.0446 (13)	0.0418 (15)	0.0509 (15)	0.0050 (11)	−0.0038 (11)	0.0199 (12)
C6	0.0429 (13)	0.0375 (15)	0.0512 (15)	0.0086 (10)	−0.0028 (11)	0.0112 (12)
C7	0.0617 (18)	0.056 (2)	0.074 (2)	0.0209 (14)	0.0040 (15)	0.0143 (16)
C8	0.067 (2)	0.077 (2)	0.087 (2)	0.0355 (18)	0.0144 (18)	0.0062 (19)
C9	0.072 (2)	0.092 (3)	0.067 (2)	0.0245 (19)	0.0255 (17)	0.0105 (19)
C10	0.0638 (18)	0.075 (2)	0.0558 (18)	0.0204 (15)	0.0142 (14)	0.0212 (16)
C11	0.0394 (13)	0.0395 (15)	0.0571 (16)	0.0132 (11)	0.0066 (11)	0.0122 (12)
C12	0.0505 (14)	0.0485 (16)	0.0451 (15)	0.0105 (12)	0.0033 (11)	0.0152 (12)
C13	0.087 (2)	0.075 (2)	0.072 (2)	−0.0207 (18)	−0.0090 (18)	0.0188 (19)
C14	0.100 (2)	0.068 (2)	0.0512 (18)	0.0160 (18)	0.0092 (17)	0.0162 (16)
C15	0.0376 (12)	0.0501 (16)	0.0510 (16)	0.0179 (11)	0.0052 (11)	0.0171 (12)
C16	0.0454 (14)	0.0471 (17)	0.074 (2)	0.0128 (12)	0.0026 (13)	0.0238 (15)
C17	0.086 (2)	0.057 (2)	0.079 (2)	0.0123 (17)	−0.0157 (18)	0.0064 (17)
C18	0.073 (2)	0.092 (3)	0.139 (4)	0.003 (2)	0.026 (2)	0.051 (3)
C19	0.0375 (13)	0.064 (2)	0.0428 (14)	0.0189 (12)	0.0092 (10)	0.0197 (13)
C20	0.0494 (15)	0.065 (2)	0.0438 (15)	0.0121 (13)	0.0142 (11)	0.0150 (13)
C21	0.094 (2)	0.110 (3)	0.0468 (18)	0.028 (2)	0.0213 (17)	0.0252 (18)
C22	0.112 (3)	0.066 (2)	0.093 (3)	0.017 (2)	0.056 (2)	0.014 (2)
C23	0.0335 (12)	0.0405 (15)	0.0653 (18)	0.0013 (10)	0.0158 (11)	0.0151 (13)
C24	0.0419 (13)	0.0495 (17)	0.0496 (15)	0.0049 (11)	0.0078 (11)	0.0132 (12)
C25	0.074 (2)	0.096 (3)	0.059 (2)	0.0237 (18)	0.0168 (16)	0.0200 (18)
C26	0.081 (2)	0.0507 (19)	0.078 (2)	0.0134 (16)	0.0142 (17)	0.0112 (16)
C27	0.0356 (12)	0.0456 (15)	0.0399 (13)	0.0048 (10)	0.0069 (10)	0.0122 (11)
C28	0.0376 (13)	0.0506 (17)	0.0583 (17)	0.0032 (11)	0.0034 (11)	0.0096 (13)
C29	0.0515 (18)	0.090 (3)	0.123 (3)	−0.0099 (17)	0.0061 (18)	0.055 (2)
C30	0.0545 (17)	0.102 (3)	0.063 (2)	0.0082 (17)	−0.0100 (14)	0.0282 (19)
C31	0.0364 (12)	0.0514 (16)	0.0473 (15)	0.0054 (11)	0.0117 (11)	0.0130 (12)
C32	0.0608 (17)	0.0503 (18)	0.082 (2)	0.0198 (13)	0.0306 (15)	0.0353 (15)
C33	0.136 (4)	0.147 (4)	0.112 (3)	0.088 (3)	0.034 (3)	0.074 (3)
C34	0.086 (2)	0.0485 (19)	0.099 (3)	0.0232 (16)	0.044 (2)	0.0212 (17)
C35	0.0458 (14)	0.0542 (17)	0.0558 (16)	0.0125 (12)	0.0093 (12)	0.0268 (13)
C36	0.0621 (17)	0.068 (2)	0.0588 (18)	0.0252 (15)	0.0103 (14)	0.0356 (15)
C37	0.078 (2)	0.062 (2)	0.0641 (19)	0.0239 (15)	0.0253 (16)	0.0417 (16)
C38	0.0586 (16)	0.0459 (16)	0.0586 (17)	0.0112 (12)	0.0197 (13)	0.0278 (13)
C39	0.0440 (13)	0.0320 (13)	0.0468 (14)	0.0114 (10)	0.0157 (10)	0.0161 (11)
C40	0.0407 (12)	0.0327 (13)	0.0465 (14)	0.0084 (10)	0.0152 (10)	0.0119 (11)
C41	0.0480 (14)	0.0439 (16)	0.0611 (17)	0.0027 (11)	0.0158 (13)	0.0187 (13)
C42	0.0419 (14)	0.0586 (19)	0.0641 (18)	−0.0048 (12)	0.0083 (13)	0.0116 (14)
C43	0.0462 (15)	0.073 (2)	0.0483 (16)	0.0022 (13)	0.0031 (12)	0.0159 (14)
C44	0.0429 (14)	0.0661 (19)	0.0472 (15)	0.0032 (12)	0.0084 (11)	0.0243 (13)

### Geometric parameters (Å, °)

**Table:**

Ce—O10	2.3947 (19)	C13—H13B	0.9600
Ce—O6	2.4169 (17)	C13—H13C	0.9600
Ce—O12	2.4174 (18)	C14—H14A	0.9300
Ce—O2	2.4376 (18)	C14—H14B	0.9300
Ce—O4	2.4404 (18)	C15—C16	1.493 (4)
Ce—O14	2.4681 (18)	C16—C17	1.353 (4)
Ce—O7	2.6115 (18)	C16—C18	1.460 (4)
Ce—O8	2.6165 (18)	C17—H17A	0.9300
Co1—O5	2.0130 (18)	C17—H17B	0.9300
Co1—O3	2.0424 (18)	C18—H18A	0.9600
Co1—N1	2.084 (2)	C18—H18B	0.9600
Co1—O1	2.0881 (19)	C18—H18C	0.9600
Co1—N2	2.134 (2)	C19—C20	1.504 (4)
Co2—O13	2.0017 (16)	C20—C22	1.331 (4)
Co2—O11	2.0352 (18)	C20—C21	1.464 (4)
Co2—O15	2.0715 (17)	C21—H21A	0.9600
Co2—N4	2.0793 (18)	C21—H21B	0.9600
Co2—N5	2.1443 (19)	C21—H21C	0.9600
O1—C11	1.251 (3)	C22—H22A	0.9300
O2—C11	1.262 (3)	C22—H22B	0.9300
O3—C15	1.249 (3)	C23—C24	1.490 (4)
O4—C15	1.255 (3)	C24—C25	1.328 (4)
O5—C19	1.251 (3)	C24—C26	1.467 (4)
O6—C19	1.255 (3)	C25—H25A	0.9300
O7—N3	1.267 (3)	C25—H25B	0.9300
O8—N3	1.248 (3)	C26—H26A	0.9600
O9—N3	1.226 (3)	C26—H26B	0.9600
O10—C23	1.256 (3)	C26—H26C	0.9600
O11—C23	1.253 (3)	C27—C28	1.499 (3)
O12—C27	1.243 (3)	C28—C29	1.359 (4)
O13—C27	1.254 (3)	C28—C30	1.433 (4)
O14—C31	1.243 (3)	C29—H29A	0.9300
O15—C31	1.245 (3)	C29—H29B	0.9300
N1—C1	1.339 (3)	C30—H30A	0.9600
N1—C5	1.341 (3)	C30—H30B	0.9600
N2—C10	1.334 (3)	C30—H30C	0.9600
N2—C6	1.344 (3)	C31—C32	1.497 (4)
N4—C35	1.340 (3)	C32—C33	1.361 (5)
N4—C39	1.347 (3)	C32—C34	1.441 (4)
N5—C44	1.336 (3)	C33—H33A	0.9300
N5—C40	1.346 (3)	C33—H33B	0.9300
C1—C2	1.369 (4)	C34—H34A	0.9600
C1—H1	0.9300	C34—H34B	0.9600
C2—C3	1.367 (5)	C34—H34C	0.9600
C2—H2	0.9300	C35—C36	1.379 (4)
C3—C4	1.376 (4)	C35—H35	0.9300
C3—H3	0.9300	C36—C37	1.373 (4)
C4—C5	1.387 (4)	C36—H36	0.9300
C4—H4	0.9300	C37—C38	1.375 (4)
C5—C6	1.479 (4)	C37—H37	0.9300
C6—C7	1.390 (4)	C38—C39	1.382 (3)
C7—C8	1.367 (4)	C38—H38	0.9300
C7—H7	0.9300	C39—C40	1.479 (3)
C8—C9	1.367 (5)	C40—C41	1.389 (3)
C8—H8	0.9300	C41—C42	1.372 (4)
C9—C10	1.375 (4)	C41—H41	0.9300
C9—H9	0.9300	C42—C43	1.374 (4)
C10—H10	0.9300	C42—H42	0.9300
C11—C12	1.490 (4)	C43—C44	1.373 (4)
C12—C14	1.346 (4)	C43—H43	0.9300
C12—C13	1.455 (4)	C44—H44	0.9300
C13—H13A	0.9600		
			
Cg1···Cg1^i^	3.816 (8)	Cg2···Cg2^ii^	3.756 (8)
			
O10—Ce—O6	91.79 (8)	C12—C13—H13B	109.5
O10—Ce—O12	89.98 (7)	H13A—C13—H13B	109.5
O6—Ce—O12	143.18 (7)	C12—C13—H13C	109.5
O10—Ce—O2	166.37 (6)	H13A—C13—H13C	109.5
O6—Ce—O2	88.83 (7)	H13B—C13—H13C	109.5
O12—Ce—O2	97.67 (7)	C12—C14—H14A	120.0
O10—Ce—O4	90.23 (7)	C12—C14—H14B	120.0
O6—Ce—O4	72.74 (6)	H14A—C14—H14B	120.0
O12—Ce—O4	144.04 (7)	O3—C15—O4	124.7 (2)
O2—Ce—O4	76.95 (7)	O3—C15—C16	117.8 (2)
O10—Ce—O14	78.17 (8)	O4—C15—C16	117.5 (2)
O6—Ce—O14	143.45 (7)	C17—C16—C18	124.2 (3)
O12—Ce—O14	72.62 (7)	C17—C16—C15	118.3 (3)
O2—Ce—O14	93.31 (8)	C18—C16—C15	117.4 (3)
O4—Ce—O14	72.25 (7)	C16—C17—H17A	120.0
O10—Ce—O7	120.53 (7)	C16—C17—H17B	120.0
O6—Ce—O7	73.78 (7)	H17A—C17—H17B	120.0
O12—Ce—O7	73.76 (6)	C16—C18—H18A	109.5
O2—Ce—O7	72.66 (6)	C16—C18—H18B	109.5
O4—Ce—O7	134.65 (6)	H18A—C18—H18B	109.5
O14—Ce—O7	141.25 (7)	C16—C18—H18C	109.5
O10—Ce—O8	71.94 (7)	H18A—C18—H18C	109.5
O6—Ce—O8	74.02 (6)	H18B—C18—H18C	109.5
O12—Ce—O8	71.65 (7)	O5—C19—O6	124.9 (2)
O2—Ce—O8	121.17 (6)	O5—C19—C20	117.0 (2)
O4—Ce—O8	141.52 (7)	O6—C19—C20	118.1 (3)
O14—Ce—O8	132.74 (7)	C22—C20—C21	123.5 (3)
O7—Ce—O8	48.59 (7)	C22—C20—C19	120.1 (3)
O5—Co1—O3	89.76 (8)	C21—C20—C19	116.4 (3)
O5—Co1—N1	94.40 (8)	C20—C21—H21A	109.5
O3—Co1—N1	101.83 (8)	C20—C21—H21B	109.5
O5—Co1—O1	96.44 (8)	H21A—C21—H21B	109.5
O3—Co1—O1	161.94 (8)	C20—C21—H21C	109.5
N1—Co1—O1	94.60 (8)	H21A—C21—H21C	109.5
O5—Co1—N2	169.14 (8)	H21B—C21—H21C	109.5
O3—Co1—N2	85.59 (8)	C20—C22—H22A	120.0
N1—Co1—N2	77.00 (8)	C20—C22—H22B	120.0
O1—Co1—N2	90.93 (8)	H22A—C22—H22B	120.0
O13—Co2—O11	95.51 (7)	O11—C23—O10	123.8 (3)
O13—Co2—O15	96.45 (7)	O11—C23—C24	117.1 (2)
O11—Co2—O15	158.37 (8)	O10—C23—C24	119.1 (3)
O13—Co2—N4	95.64 (7)	C25—C24—C26	123.3 (3)
O11—Co2—N4	97.37 (8)	C25—C24—C23	119.6 (3)
O15—Co2—N4	99.30 (7)	C26—C24—C23	117.2 (2)
O13—Co2—N5	172.77 (7)	C24—C25—H25A	120.0
O11—Co2—N5	85.03 (7)	C24—C25—H25B	120.0
O15—Co2—N5	85.35 (7)	H25A—C25—H25B	120.0
N4—Co2—N5	77.15 (7)	C24—C26—H26A	109.5
C11—O1—Co1	101.33 (16)	C24—C26—H26B	109.5
C11—O2—Ce	145.62 (16)	H26A—C26—H26B	109.5
C15—O3—Co1	124.83 (16)	C24—C26—H26C	109.5
C15—O4—Ce	138.76 (16)	H26A—C26—H26C	109.5
C19—O5—Co1	124.84 (16)	H26B—C26—H26C	109.5
C19—O6—Ce	144.81 (19)	O12—C27—O13	123.9 (2)
N3—O7—Ce	96.76 (15)	O12—C27—C28	118.8 (2)
N3—O8—Ce	97.03 (15)	O13—C27—C28	117.3 (2)
C23—O10—Ce	140.38 (18)	C29—C28—C30	123.9 (3)
C23—O11—Co2	119.12 (17)	C29—C28—C27	118.5 (3)
C27—O12—Ce	157.24 (18)	C30—C28—C27	117.6 (2)
C27—O13—Co2	123.64 (15)	C28—C29—H29A	120.0
C31—O14—Ce	161.91 (19)	C28—C29—H29B	120.0
C31—O15—Co2	110.43 (16)	H29A—C29—H29B	120.0
C1—N1—C5	118.7 (2)	C28—C30—H30A	109.5
C1—N1—Co1	124.28 (17)	C28—C30—H30B	109.5
C5—N1—Co1	116.93 (16)	H30A—C30—H30B	109.5
C10—N2—C6	118.8 (2)	C28—C30—H30C	109.5
C10—N2—Co1	125.82 (19)	H30A—C30—H30C	109.5
C6—N2—Co1	115.00 (16)	H30B—C30—H30C	109.5
O9—N3—O8	121.6 (3)	O14—C31—O15	122.4 (3)
O9—N3—O7	120.8 (3)	O14—C31—C32	119.5 (3)
O8—N3—O7	117.5 (2)	O15—C31—C32	117.9 (2)
C35—N4—C39	118.9 (2)	C33—C32—C34	123.5 (3)
C35—N4—Co2	124.13 (16)	C33—C32—C31	118.4 (3)
C39—N4—Co2	117.00 (15)	C34—C32—C31	118.0 (3)
C44—N5—C40	118.8 (2)	C32—C33—H33A	120.0
C44—N5—Co2	126.25 (16)	C32—C33—H33B	120.0
C40—N5—Co2	114.96 (15)	H33A—C33—H33B	120.0
N1—C1—C2	122.5 (3)	C32—C34—H34A	109.5
N1—C1—H1	118.8	C32—C34—H34B	109.5
C2—C1—H1	118.8	H34A—C34—H34B	109.5
C3—C2—C1	119.1 (3)	C32—C34—H34C	109.5
C3—C2—H2	120.5	H34A—C34—H34C	109.5
C1—C2—H2	120.5	H34B—C34—H34C	109.5
C2—C3—C4	119.3 (3)	N4—C35—C36	122.6 (2)
C2—C3—H3	120.4	N4—C35—H35	118.7
C4—C3—H3	120.4	C36—C35—H35	118.7
C3—C4—C5	119.1 (3)	C37—C36—C35	118.4 (3)
C3—C4—H4	120.5	C37—C36—H36	120.8
C5—C4—H4	120.5	C35—C36—H36	120.8
N1—C5—C4	121.3 (3)	C36—C37—C38	119.5 (2)
N1—C5—C6	115.3 (2)	C36—C37—H37	120.3
C4—C5—C6	123.3 (2)	C38—C37—H37	120.3
N2—C6—C7	121.3 (3)	C37—C38—C39	119.6 (2)
N2—C6—C5	115.1 (2)	C37—C38—H38	120.2
C7—C6—C5	123.5 (2)	C39—C38—H38	120.2
C8—C7—C6	119.0 (3)	N4—C39—C38	121.0 (2)
C8—C7—H7	120.5	N4—C39—C40	115.50 (19)
C6—C7—H7	120.5	C38—C39—C40	123.5 (2)
C9—C8—C7	119.6 (3)	N5—C40—C41	120.7 (2)
C9—C8—H8	120.2	N5—C40—C39	115.3 (2)
C7—C8—H8	120.2	C41—C40—C39	124.0 (2)
C8—C9—C10	119.0 (3)	C42—C41—C40	119.5 (2)
C8—C9—H9	120.5	C42—C41—H41	120.3
C10—C9—H9	120.5	C40—C41—H41	120.3
N2—C10—C9	122.3 (3)	C41—C42—C43	119.9 (2)
N2—C10—H10	118.8	C41—C42—H42	120.1
C9—C10—H10	118.8	C43—C42—H42	120.1
O1—C11—O2	120.3 (2)	C42—C43—C44	117.8 (3)
O1—C11—C12	118.9 (2)	C42—C43—H43	121.1
O2—C11—C12	120.8 (2)	C44—C43—H43	121.1
C14—C12—C13	123.4 (3)	N5—C44—C43	123.4 (2)
C14—C12—C11	119.8 (3)	N5—C44—H44	118.3
C13—C12—C11	116.8 (2)	C43—C44—H44	118.3
C12—C13—H13A	109.5		

Symmetry codes: (i) −*x*+2, −*y*+1, −*z*+1; (ii) −*x*+2, −*y*+2, −*z*.

### Hydrogen-bond geometry (Å, °)

**Table:**

Cg1 and Cg2 are the centroids of the N1/N2/C1–C10 and N4/N5/C35–C40 rings, respectively.

**Table:**

*D*—H···*A*	*D*—H	H···*A*	*D*···*A*	*D*—H···*A*
C2—H2···O7^iii^	0.93	2.59	3.322 (4)	136
C3—H3···O9^iii^	0.93	2.45	3.307 (5)	154
C7—H7···O3^i^	0.93	2.58	3.452 (4)	156
C29—H29B···O12	0.93	2.43	2.750 (4)	100
C37—H37···O9^iv^	0.93	2.51	3.364 (4)	152

Symmetry codes: (iii) −*x*+1, −*y*+1, −*z*+1; (i) −*x*+2, −*y*+1, −*z*+1; (iv) −*x*+1, −*y*+2, −*z*.

## References

[bb1] Farrugia, L. J. (1997). *J. Appl. Cryst.* **30**, 565.

[bb2] Higashi, T. (1995). *ABSCOR* Rigaku Corporation, Tokyo, Japan.

[bb3] Lu, W.-M., Wu, J.-B., Dong, N., Chun, W.-G., Gu, J.-M. & Liang, K.-L. (1995). *Acta Cryst.* C**51**, 1568–1570.

[bb4] Rigaku (1998). *RAPID-AUTO* Rigaku Corporation, Tokyo, Japan.

[bb5] Rigaku/MSC (2002). *CrystalStructure* Rigaku and Rigaku/MSC, The Woodlands Texas, USA.

[bb6] Sheldrick, G. M. (2008). *Acta Cryst.* A**64**, 112–122. 10.1107/S0108767307043930 18156677

[bb7] Spek, A. L. (2009). *Acta Cryst.* D**65**, 148–155. 10.1107/S090744490804362X PMC2631630 19171970

[bb8] Wu, B. & Guo, Y. (2004). *Acta Cryst.* E**60**, m1356–m1358.

[bb9] Zhu, Y., Lu, W.-M. & Chen, F. (2004*a*). *Acta Cryst.* E**60**, m963–m965.

[bb10] Zhu, Y., Lu, W. & Chen, F. (2004*b*). *Acta Cryst.* E**60**, m1459–m1461.

[bb11] Zhu, Y., Lu, W.-M., Ma, M. & Chen, F. (2005). *Acta Cryst.* E**61**, m1610–m1612.

